# Nanoparticle-Based Treatment in Glioblastoma

**DOI:** 10.3390/jpm13091328

**Published:** 2023-08-29

**Authors:** Diogo Roque, Nuno Cruz, Hugo Alexandre Ferreira, Catarina Pinto Reis, Nuno Matela, Manuel Herculano-Carvalho, Rita Cascão, Claudia C. Faria

**Affiliations:** 1 Department of Neurosurgery, Hospital de Santa Maria, Centro Hospitalar Universitário Lisboa Norte (CHULN), 1649-028 Lisbon, Portugal; luis.diogo.roque@gmail.com (D.R.); mirro170564@gmail.com (M.H.-C.); 2 Instituto de Medicina Molecular João Lobo Antunes, Faculdade de Medicina, Universidade de Lisboa, 1649-028 Lisboa, Portugal; ritacascao@medicina.ulisboa.pt; 3 Instituto de Biofísica e Engenharia Biomédica, IBEB, Faculdade de Ciências, Universidade de Lisboa, 1749-016 Lisboa, Portugal; nunocruz9009@gmail.com (N.C.); hugoferreira@campus.ul.pt (H.A.F.); catarinareis@ff.ulisboa.pt (C.P.R.); nmatela@ciencias.ulisboa.pt (N.M.); 4 iMED.ULisboa, Research Institute for Medicines, Faculdade de Farmácia, Universidade de Lisboa, Av. Prof. Gama Pinto, 1649-003 Lisboa, Portugal; 5 Clínica Universitária de Neurocirurgia, Faculdade de Medicina da Universidade de Lisboa, 1649-028 Lisboa, Portugal

**Keywords:** glioblastoma, nanoparticles, blood-brain barrier, therapy delivery

## Abstract

Glioblastoma (GB) is a malignant glioma associated with a mean overall survival of 12 to 18 months, even with optimal treatment, due to its high relapse rate and treatment resistance. The standardized first-line treatment consists of surgery, which allows for diagnosis and cytoreduction, followed by stereotactic fractionated radiotherapy and chemotherapy. Treatment failure can result from the poor passage of drugs through the blood–brain barrier (BBB). The development of novel and more effective therapeutic approaches is paramount to increasing the life expectancy of GB patients. Nanoparticle-based treatments include epitopes that are designed to interact with specialized transport systems, ultimately allowing the crossing of the BBB, increasing therapeutic efficacy, and reducing systemic toxicity and drug degradation. Polymeric nanoparticles have shown promising results in terms of precisely directing drugs to the brain with minimal systemic side effects. Various methods of drug delivery that pass through the BBB, such as the stereotactic injection of nanoparticles, are being actively tested in vitro and in vivo in animal models. A significant variety of pre-clinical studies with polymeric nanoparticles for the treatment of GB are being conducted, with only a few nanoparticle-based drug delivery systems to date having entered clinical trials. Pre-clinical studies are key to testing the safety and efficacy of these novel anticancer therapies and will hopefully facilitate the testing of the clinical validity of this promising treatment method. Here we review the recent literature concerning the most frequently reported types of nanoparticles for the treatment of GB.

## 1. Introduction

Primary malignant neoplasms of the brain, such as glioblastoma (GB) (World Health Organization (WHO) grade 4), are associated with a poor prognosis. The mean overall survival is 12 to 18 months in patients undergoing maximal safe resection followed by adjuvant radiation and chemotherapy with temozolomide (TMZ) [1]. The mean overall 5-year survival rate is lower than 6% [2]. The major concern in GB is its notable treatment resistance and high probability of relapse, with glioma stem cells (GSC) contributing to tumor initiation, regrowth, and therapeutic resistance mechanisms. Recurrent GB is usually not amenable to surgical resection due to tumor infiltration and invasion of nearby brain structures [3].

The treatment of GB has evolved over the past decades. Some adjuvant treatment options have been developed, namely locally delivered drugs, such as carmustine depots (Gliadel^®^), which release therapeutic carmustine concentrations until 120 h of the post-operative period [4], and systemically delivered chemotherapy, such as temozolomide and nitrosoureas. Unfortunately, nitrosoureas (e.g., carmustine) are associated with considerable adverse effects, including bone marrow suppression, hepatotoxicity, nephrotoxicity, and interstitial lung disease. For this reason, they are mainly used in the treatment of recurrent GB [5].

This review focus on the most recent data concerning the development of nanoparticle technology and discusses its potential for the treatment of patients with GB.

## 2. Current GB Treatment and Its Challenges

The standard of care treatment for GB patients consists of a multimodal approach based on maximally safe surgical resection (to allow cytoreduction of the tumor, obtain histological diagnosis, and address the potential life-threatening mass effect and edema of the tumor) followed by radiotherapy and chemotherapy [6].

Surgical resection is usually the first phase of treatment when a GB patient has a good performance status (a Karnofsky performance status of 60% or more is generally considered favorable in the clinical setting). Surgical resection of the tumor improves symptoms and neurological defects, reducing the mass effect and the associated peritumoral edema. Furthermore, surgery allows tumor sampling for histopathological and genetic characterization, which will influence the radiotherapeutic and chemotherapeutic regimen to be chosen [6].

Adjuvant treatment for GB following maximal safe resection was standardized after the work of Roger Stupp and colleagues. In this study, 573 patients from 85 centers were randomized into adjuvant treatment with radiotherapy alone or treatment with radiotherapy and chemotherapy with TMZ. This resulted in improved median survival of patients in the combined treatment group, with minimal additional toxicity [7]. As a result of this study, patients with GB are treated with radiotherapy and TMZ after surgical resection.

More recently, tumor-treating fields (TTF) emerged as a treatment modality that selectively promotes an antimitotic effect on GB cells using a headset that generates a low-intensity, intermediate-frequency (200 Hz), alternating electric field, leading to replication cycle arrest and apoptosis. Roger Stupp and colleagues conducted a randomized clinical trial that included 695 patients who were submitted to surgical resection or biopsy followed by concomitant radiochemotherapy. Patients were randomized into two groups: TTF plus maintenance TMZ or TMZ alone. Progression-free survival (6.7 vs. 4.0 months) and overall survival (20.9 vs. 16.0 months) were superior in the TTF-TMZ group when compared to TMZ alone, respectively [8].

### 2.1. The Blood–Brain Barrier

One of the main difficulties in the treatment of this aggressive brain tumor is the poor passage of the drugs through the blood–brain barrier (BBB). The BBB is a physically and biochemically selective barrier that protects the brain but also inhibits the passive penetration of hydrophilic molecules from the circulation into the central nervous system (CNS). This barrier is a complex structure composed of endothelial cells, pericytes, and astrocytes, and the endothelium is connected by tight junctions that control its permeability. Under physiological conditions, these tight junctions are highly regulated. In GB, these junctions are not as tightly connected, and the BBB becomes ‘leaky’. Some authors name this altered BBB the blood–brain tumor barrier (BBTB) [9]. Clinical studies indicate that most GB patients present an intact BBB in certain brain regions. The intact barrier is capable of blocking the delivery of anticancer agents, preventing the diffusion of 98% of small molecules and 100% of large molecules into the brain [10]. For this reason, the route of delivery is also an aspect that needs to be investigated and fine-tuned to improve treatment efficacy in patients with GB.

One possibility to overcome BBB selectivity is the development of a specialized transport system that facilitates the passage of molecules and their targeted delivery into the brain. In recent years, nanotechnology-based systems have gained relevance as promising effective therapeutic agents against cancer cells, including GB, mostly in animal models [3]. This technology aims not only to increase transport through the BBB, with the expected effect of improving therapeutic efficacy, but also to reduce systemic toxicity and drug degradation [11]. Nanotechnology-based systems most often involve the use of nanoparticles (NP) coated with specific ligands for BBB receptors with the goal of enabling the targeted delivery of drugs, as well as polyethylene glycol (PEG) to improve circulation time by preventing engulfment by the mononuclear phagocyte system [9,12].

### 2.2. Spatiotemporal Heterogeneity in GB

Cancer progression, namely dissemination and resistance to therapy, is enabled by tumor evolution, with the selection of subclones with different genotypes and phenotypes. It is fundamental to study multiple spatiotemporal tumor samples from the same patient to gain insight about cancer evolution and identify and characterize subclones of cancer cells to develop better targeted therapies against their specific properties. GB is characterized by a high degree of phenotypic and genetic heterogeneity, therapeutic resistance, and relapse. Therefore, studies on spatiotemporal heterogeneity are crucial in this context because it represents a challenge for treatment success but also an opportunity for developing novel therapeutic strategies. Interactions over time and across different locations between cancer and immune cells generate an immunosuppressive tumor microenvironment that contributes to treatment failure. On the other hand, a better characterization of this spatiotemporal heterogeneity allows the identification of new targets for therapy. For instance, a study comparing paired primary and recurrent GB tumors identified shared somatic mutations that could be biologically relevant and promising targets for therapy [13]. Another study evaluated the spatiotemporal dynamics of glioma stem cell (GSC) invasion, a relevant feature in GB. Elucidation of these mechanisms may support the identification of more effective therapeutic targets to be tested in GB [14]. Recently, an interesting review from Comba and colleagues very nicely summarized this spatiotemporal heterogeneity of gliomas and their impact on treatment [15]. The use of NP could be a useful strategy to deliver specific therapies against relevant target cancer subclones and to deliver distinct therapeutic modalities to different tumor areas with divergent characteristics (edge- vs. core-located tumor cells).

### 2.3. Therapeutic Delivery in GB

Recent studies specifically evaluated the systemic targeting of GB using nanoparticles and their capacity to selectively deliver therapies to target cells. Both are crucial aspects in the context of brain tumors, as in the case of GB. Intravenous injection is the least invasive drug delivery route to the brain, but drugs must be able to effectively cross the BBB. Proteins and viral particles can be transported and targeted through this barrier.

Gregory et al. have engineered a GB-targeting synthetic protein nanoparticle (SPNP) with albumin, loaded with the cell-penetrating peptide iRGD and the siRNA against *signal transducer and activation of transcription 3 factor* (*STAT3i*), known to be associated with GB progression. Albumins engage with cell-surface receptors overexpressed in glioma cells, and the iRGD allows BBB penetration and distribution throughout the tumor mass. The effective systemic delivery of these protein nanoparticles downregulated *STAT3*, reduced tumor progression, and extended survival in GB mouse models when combined with the standard of care therapy (ionized radiation) [16].

Alghamri et al. also tested the ability of SPNPs to deliver their therapeutic content to GB tumors after systemic delivery. In this study, an adjuvant CXCR4 inhibitor was encapsulated in iRGD-coated SPNPs to suppress the CXCL12/CXCR4 signaling pathway, which is activated in GB and associated with tumor progression. Blockade of this signaling pathway by SPNPs resulted in inhibition of GB proliferation, sensitization to radiation therapy, and reduced infiltration of immune-suppressive cells. Combined SPNP and radiation treatment improved mouse survival [17].

Immunotherapeutic approaches, such as CAR T cells, have been attempted in GB treatment, but their effectiveness has also been limited due to the BBB. Curiously, neutrophils can cross this barrier. Chang and colleagues reported that tumor microenvironment-responsive nanodrugs delivered by CAR neutrophils can target GB, exhibiting superior and specific anti-tumor activities, reducing off-target effects, and increasing the lifespan in mouse models. The data suggested that >20% of administered nanodrugs are delivered to the tumor mass by CAR neutrophils, in contrast to 1% reached by free nanodrugs. One of the challenges in treating GB patients is the development of therapeutic resistance mechanisms, such as the upregulation of CD47 and PD-L1 [18]. Interestingly, Zhang et al. generated a dual CD47/PD-L1-targeting nanoparticle loaded with a stimulator of interferon genes (STING) agonist. These nanoparticles specifically engage tumor-associated myeloid cells (TAMCs) with GB cells via the dual anti-CD47/PD-L1 ligation. Activation of STING in target TAMCs induces the production of pro-inflammatory cytokines, which stimulate infiltration and activation of effector T cells. This approach of blocking innate (CD47) and effector (PD-L1) checkpoint molecules while delivering the STING agonist potentiates anti-tumor immunity and radiation therapy in GB treatment, promoting tumor regression in vivo [19]. Interestingly, Kozielski et al. were the first to report a multimodal approach using NP. Some authors have used a synthetic, bioreducible, biodegradable polymeric nanoparticle that is able to encapsulate and deliver different siRNA molecules simultaneously and in high amounts. Importantly, this system facilitates combinatorial therapy against diverse target genes relevant for GB progression. These NPs combining diverse anti-GB siRNAs lead to augmented GB cell death, reduced migration in vitro, and diminished tumor burden in vivo. Moreover, several authors demonstrated that the delivery of siRNA was cancer-selective, avoiding off-target side effects in healthy cells [20].

## 3. Definition and Properties of Nanoparticles (NP)

Richard Feynman introduced the notion of nanotechnology in 1959 [21], and the word “nanoparticle” was used for the first time in 1976 by Jörg Kreuter. Ten years later, the first research article based on NPs was published by Robert Gurny, but the nanotechnological era, including nanoparticle-based drug delivery systems, only started in the 2000s. In the past ten years, in particular, the rate of publications related to NP therapies has grown significantly [22].

**Figure 1:**
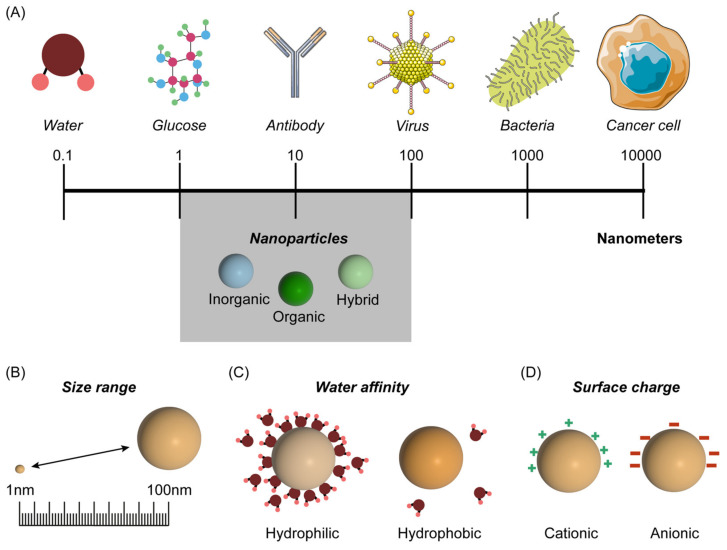
Schematic representation of nanoparticles in a spectrum of inorganic/organic molecules, macromolecules, and large molecular assemblies such as viruses and cells, measured in nanometers of different biological molecules (**A**). Distinct properties are shown as: size range (**B**); water affinity (**C**); and surface charge (**D**). Parts of the figure were drawn using pictures from Servier Medical Art. Servier Medical Art by Servier is licensed under a Creative Commons Attribution 3.0 Unported License (https://creativecommons.org/licenses/by/3.0/, accessed on 17 March 2023).

Various definitions of NP are employed in the literature (with slight differences), but it is usually defined as a nanomaterial of 1 to 100 nm in diameter [12] (Figure 1A). NPs can be classified as organic-based, inorganic-based, and hybrid [12].

This technology allows for passive or active drug transport and delivery to tumors [3]. Passive drug delivery is based on the observation that certain-sized particles show tropism towards tumors due to BBB disruption in the tumor area, known as the enhanced penetration and retention (EPR) effect [23], which is actively debated for its relevance and applications in nanomedicine/nanotechnology-based tumor treatment [24]. Nevertheless, it is crucial to have an active transport system for drug delivery, particularly in areas where the BBB remains intact. This type of drug delivery is attained by designing NPs with glial tissue- and/or BBB-specific ligands on their surface [3].

The pharmacokinetics of a nanoformulation vary according to size (Figure 1A,B), hydrophobicity (Figure 1C), and surface charge of the NP (Figure 1D). In terms of size, if the particle is smaller than 10 nm, the clearance by renal glomerular filtration will significantly reduce its serum half-life and, consequently, its efficiency [25]. Contrarily, large NPs with diameters above 150 nm are prone to rapid clearance by the reticuloendothelial system (RES), which would imply a larger dose to achieve adequate therapeutic levels and result in increased systemic side effects [25]. One possibility to solve this issue is to add a PEG coating to the NP (PEGylation of the NP), reducing its clearance by the RES, and thus increasing its serum half-life [12]. Regarding water affinity, hydrophilic NPs have an increased half-life in circulation, but their capacity to cross the BBB is reduced. On the contrary, hydrophobic NPs can cross the BBB but are more susceptible to RES clearance due to their reduced half-life. Amphiphilic NPs, with both hydrophilic and hydrophobic properties, combine features that augment their BBB-crossing capacity and serum half-life [25]. In terms of surface charge, anionic particles have improved diffusion within the interstitium but show reduced brain tumor cellular uptake compared to cationic particles, which tend to cross the BBB more readily [25,26]. Neutral charges facilitate spread through the tumor’s extracellular matrix [25].

Different properties of NPs, like their morphology, surface charge, and components, influence their capacity to cross the BBB, serum half-life, and ability to encapsulate certain types of drugs.

## 4. Types of Nanoparticles

**Figure 2:**
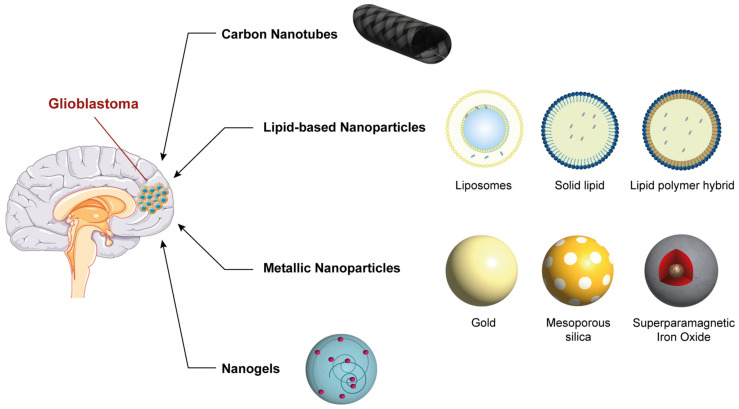
Representation of the main types of nanoparticles being studied as potential candidates for the treatment of glioblastoma. Parts of the figure were drawn using pictures from Servier Medical Art. Servier Medical Art by Servier is licensed under a Creative Commons Attribution 3.0 Unported License (https://creativecommons.org/licenses/by/3.0/, accessed on 17 March 2023).

In the following section, we describe the major types of nanoparticles being actively studied as potential candidates for the treatment of malignant brain tumors such as glioblastoma (Figure 2).

### 4.1. Lipid-Based Nanoparticles

Lipid-based NPs include liposomes, solid lipid NPs, and lipid-polymer hybrid NPs. These types of NPs have already shown promising results in the treatment of GB in preclinical studies [26,27].

#### 4.1.1. Liposomes

Liposomes are closed bilayer structures formed by phospholipids with a phosphate polar head and a hydrophobic lipid tail, making these structures capable of transporting encapsulated drugs regardless of their physicochemical properties. Drugs can be incorporated in the hydrophobic lipid tails or in the hydrophilic polar head [26,28]. Liposomes are classified according to the number of phospholipid bilayers as unilamellar or multilamellar vesicles [26]. Unilamellar vesicles are further subdivided, based on their size, into small unilamellar vesicles (<100 nm), large unilamellar vesicles (100 nm to 1 µm), and giant unilamellar vesicles (>1 µm) [28]. Liposomes can be incorporated with hydrophilic and hydrophobic drugs to treat cancer, as immunomodulatory agents, and even for genetic therapies [28].

The main advantages of liposomes are their biocompatibility, their capacity for encapsulating different types of molecules (both polar and non-polar), and the protection they confer to the encapsulated drug against external agents [26,28]. The limitations associated with liposomes are their short half-life in circulation and their tendency for rapid clearance by the RES. A way of circumventing this rapid clearance and augmenting treatment effectiveness is through PEGylation of liposomes, which prevents RES uptake [12,25,28]. Another relevant disadvantage of these NPs is related to the cost of production, which hinders the diffusion of their investigation and, consequently, stalls the understanding of their clinical applications [26].

Regarding liposome preparation, it typically involves different sequential stages. Firstly, lipids are placed in a homogeneous solvent solution at specific temperatures and concentrations. Second, the solvent is removed, and the lipids are incorporated into an aqueous solution. Third, the particles are purified and characterized. The next phase involves drug incorporation in these nanoparticles, which can be passive or active. Passive drug loading is conducted while the liposomes are being assembled and is ideal for the loading of hydrophobic drugs. On the other hand, active drug loading is executed after the liposomes have been produced and tends to be used for the encapsulation of hydrophilic (polar) drugs [26].

In the past few years, various authors have published their work on liposome-based nanoformulations for the treatment of GB. Zhang et al. developed a liposome-encapsulating doxorubicin and modified it with the cell-penetrating peptide CB5005 (DOX@CB5005@LP) to treat mice orthotopically implanted with U87 cells, resulting in prolonged overall survival after treatment [29]. Zhu et al. described a nanoformulation with a liposome modified with a ginsenoside, Rg3, encapsulating paclitaxel to increase membrane penetration (PTX@Rg3@LP) to treat C6 glioma-carrying mice. The authors demonstrated an increase in the cellular uptake of paclitaxel and in the ratio of the M1 phenotype of tumor-associated macrophages (TAMs), with antitumoral properties, over the protumoral M2 TMAs [28,30], resulting in improved survival [31].

Finally, a report from Hu Y et al. tested an intranasally delivered liposome containing a small interfering RNA (siRNA) against *c-Myc*, modified with a peptide derived from a penetratin named 89WP. This modification allowed penetration into the nasal mucosa, inducing rapid release of siRNA to promote downregulation of c-Myc mRNA and protein expression. This liposome was used to treat an orthotopic mouse model of glioma, resulting in prolonged survival by inducing apoptosis [32].

#### 4.1.2. Solid Lipid Nanoparticles

Solid lipid nanoparticles (SLNs) are composed of lipids that remain in a solid state at physiological temperatures [26]. These particles have a lipidic (hydrophobic) compact core coated by phospholipids, which can enhance the efficiency of entrapping hydrophobic drugs [26].

The mechanisms allowing for entrance of SLNs into the central nervous system are an active matter of debate [33]. Although paracellular entry of nanoparticles through transient opening of tight junctions between endothelial cells in the BBB has been advocated previously as the main pathway of nanoparticles’ availability in the brain [33,34,35], newer studies show that the predominant route is NP endocytosis [33,36].

Ak et al. developed a SLN that encapsulated carmustine and a SLN that encapsulated temozolomide, which were then used in vitro in U87MG cells. The authors showed increased antitumor activity using this treatment approach in comparison with both unencapsulated carmustine and unencapsulated temozolomide [37].

#### 4.1.3. Lipid–Polymer Hybrid Nanoparticles

Lipid–polymer hybrid nanoparticles (LPNPs) are composed of a hydrophobic polymeric core coated by a lipid layer, which increases biocompatibility, coupled with a hydrophilic layer that prolongs serum half-life and promotes the stability of the NP [26].

These NPs were initially designed to overcome some of the structural and biochemical limitations of liposomes and polymeric NPs, namely, inadequate content fixation, structural decay, and limited serum half-life [38]. By combining the features of different NPs in a hybrid particle and adding an outer steric stabilizer, like a PEG layer, LPNPs have a more prolonged time in circulation [38].

Shi et al. generated a nanoformulation with a LPNP that encapsulated docetaxel, coupled with the arginine-glycine-aspartic acid (RGD peptide), to increase the uptake by C6 glioma cells overexpressing α(v)ß3 integrin implanted in rats [39]. The result was a 3.35-fold increase in median survival in rats treated with these nanoformulations compared with animals treated with free docetaxel [39].

### 4.2. Nanogels

This class of nanoparticles is composed of cross-linked hydrophilic polymeric networks and has shown increasing promise in the past few years [28]. Nanogels possess numerous hydrophilic groups that contribute to the swelling of the NP, enlarge the interior mesh, and allow the entrapped drugs to be released by passive diffusion [28,40]. Nanogels can be fine-tuned in terms of size (ranging from 50 to 500 nm) and conformation according to the physicochemical characteristics of microenvironmental factors. Temperature, pH, ionic change, redox state, and light are used as stimuli for drug release [28,41].

The GB microenvironment is typically acidic due to excessive programmed tumor cell death and the Warburg effect (aerobic glycolysis metabolism characteristic of cancer cells) [42]. Song P. et al. created a pH/reduction-sensitive carboxymethyl chitosan nanogel (CMCSN) modified by targeting the peptide angiopep-2 (ANG) and loaded with doxorubicin (DOX@ANG@CMCSN), resulting in enhanced targeting of GB cells and reduced systemic toxicity, namely cardiotoxicity, which is a common side effect of doxorubicin treatment [43,44]. The addition of ANG to the nanoformulation augmented BBB penetration and tumor targeting in both in vitro and in vivo models. C6 tumor-bearing mice treated with the DOX@ANG@CMCSN nanoformulation showed a 2–3-fold increase in cellular uptake and antitumor activity [43].

Javed et al. utilized atom transfer radical polymerization to create lignin-g-P (NIPAM-co-DMAEMA) gold nanogels. These nanogel NPs were loaded with curcumin or piperine or co-loaded with curcumin and piperine to test their in vitro efficiency for the treatment of U251 MG cells. The glioma cells treated with the co-loaded curcumin–piperine lignin-g-P (NIPAM-co-DMAEMA) gold nanogels showed higher rates of caspase-3-induced apoptosis than the cells treated with nanogels loaded with one of the two agents alone [45].

### 4.3. Carbon Nanotubes

Carbon nanotubes (CNT) are cylindrical nanomaterials with a wide surface area for efficient drug loading and favorable penetration capacity [28,46]. These NPs can be subdivided into single-walled CNT (0.4 to 20 nm) or multi-walled CNT (2 to 100 nm) [28,47]. CNT have a high cell permeability, allowing BBB crossing, but due to their low solubility in aqueous media, these NPs require surface modifications to add hydrophilic groups/polymers to allow for adequate serum circulation [28]. Salazar et al. developed a CNT that carries carmustine for testing in malignant glioma cell lines. The carbon nanotubes loaded with carmustine showed a continuous kinetic release of the drug, with a maximal release at 72 h. This contributed to increased intratumoral drug concentration, less systemic toxicity, and equivalent normal brain cytotoxicity when compared to the free drug [48].

Villegas et al. conducted a study in which multiwalled CNTs (MWCNTs) were tested in vitro on BV2 microglial cells of an immortalized murine cell line model. In this study, it is worth mentioning that the multiwalled CNT caused severe interference in cell migration and phagocytosis [49].

### 4.4. Metallic Nanoparticles

Several metallic nanomaterials have been investigated as agents for the treatment of patients with GB [50]. These NPs can be synthesized and altered with different functional groups, which renders them versatile in terms of conjugation with different ligands and drugs [51]. The most frequently described metallic NPs are mesoporous silica NPs, gold NPs, and superparamagnetic iron oxide NPs, which are discussed in the next sections.

#### 4.4.1. Mesoporous Silica

Mesoporous silica nanoparticles (MSNs) are composed of a metal core encapsulated by a silica outer shell. Their sizes usually range from 2 to 50 nm, and these particles have favorable biocompatibility and a high surface area. Importantly, reports have demonstrated insignificant cytotoxicity with the use of these NPs [28,52]. MSNs require modifications with the addition of specific homing ligands to gain tropism towards glioma cells [28,52].

Recently, Bielecki et al. developed a personalized MSN loaded with cyclic diguanylate monophosphate (cdGMP), which is a stimulator of interferon gene (STING) agonist, with the rationale of reversing the GB-induced immunosuppression in the tumor microenvironment (TME). The immunosuppression is partly justified by the TGF-ß-rich environment, which suppresses major histocompatibility complex (MHC) expression. This immune-mediated MSN resulted in the stimulation of dendritic cell and macrophage influx through the BBB and the induction of CD8^+^ T cell activity against tumor cells while preserving the surrounding brain parenchyma. This study was conducted in vivo with female C57BL/6 albino mice [53].

A study from Zhu et al. demonstrated that treatment with a lipid-coated MSN encapsulating paclitaxel with a surface angiopep-2 (PTX@ANG@MSN) could prolong survival both in vitro and in vivo (in rats implanted with C6 glioma cells). The angiopep-2 was added to the structure to increase penetration into the brain parenchyma, and the lipid moiety was idealized for this customized NP to improve surface functionalization. Regarding the latter (addition of the lipid layer), this resulted in a 4.5-fold increase in the serum half-life of the nanoformulation when compared to that of the MSN without the lipid component [54].

#### 4.4.2. Gold Nanoparticles

Gold nanoparticles (GNPs) have distinct conformations, with core sizes ranging from 1 to 200 nm [28,55]. The physicochemical properties of GNPs (size, shape, surface coating, and surface charge) can significantly alter their biodistribution [28,56]. In particular, GNPs with a smaller size (approximately 10 nm) can readily cross the relatively intact BBB through passive diffusion [56,57]. In contrast, the BBB gets leakier in some areas near the tumor (hence the term BBTB), allowing the passage of larger GNP (in the range of 100 nm) [56]. The surface of GNPs is usually modified with the addition of hydrophilic molecules, such as PEG, to avoid rapid circulation clearance by the RES [56].

Radiosensitization of GB tumors has recently been studied with iodine, gadolinium, and gold [30]. Gold has a high atomic number; thus, it efficiently absorbs X-rays, boosting radiotherapy and conferring this type of NP with the quality of being a radiosensitizer [56].

Coluccia et al. described a cisplatin-conjugated GNP (Cis@UP@GNP) in combination with magnetic resonance imaging (MRI)-guided focused ultrasound for GB treatment. This treatment inhibited glioma cells by inducing DNA damage and apoptosis both in vitro (U251 and U87 GB cell lines) and in vivo (NOD scid gamma mice submitted to xenotransplantation of U251 GB cells) when compared to free cisplatin. Furthermore, a synergistic effect with radiotherapy was also shown [58].

#### 4.4.3. Superparamagnetic Iron Oxide Nanoparticles

Superparamagnetic iron oxide NPs (SPIONs) have a core made from magnetite (Fe_3_O_4_) and/or maghemite (γ-Fe_2_O_3_), which induces their superparamagnetic properties and allows for the targeting of the NP towards specific sites with the use of an external magnetic field. Additionally, the pattern of NP deposition in the brain tissue can be confirmed by MRI [3]. SPIONs are coated with polymers to avoid clustering and promote biocompatibility [59]. These NPs are an interesting tool for the imaging of brain tumors because they act as a negative contrast agent, making tumors appear as hypointense masses on T2-weighted MRI images [59].

In addition to the clinical interest in using SPIONs as imaging agents, their structures can also be tuned for use as drug delivery systems. Hollow SPIONs can be used for intratumoral delivery of hydrophobic drugs [59]. Zhu et al. reported a doxorubicin-carrying hollow SPION (DOX@SPION) with a hydrodynamic diameter of 192 nm for the treatment of U87 cells [41]. DOX@SPION induced a 2-fold increase in caspase-3 activity when compared to free doxorubicin, and the concentration of drug that caused a 50% cytotoxic effect was significantly lower for the nanoparticle compared with that of the free drug [60].

Due to SPION’s magnetic properties, exposure to an alternating external magnetic field causes such NPs to release energy in the form of heat, causing necrosis of the tumor tissue. This process is known as magnetic-particle-mediated hyperthermia [31,40]. Moreover, it is worth mentioning that SPION allows combined chemotherapy and hyperthermia, which can be advantageous in GB treatment [59].

## 5. Clinical Trials with Nanoparticles in GB

Table 1 summarizes some nanoparticle delivery systems that have entered clinical trials for the treatment of GB [61,62,63,64,65,66,67]. Aparicio-Blanco J et al. reviewed the most relevant nanoparticle-based drug delivery systems tested in the treatment of glioblastoma [68].

## 6. Conclusions and Future Perspectives

Nanotechnology-based drug delivery systems have been extensively studied in the past few years. Due to improvements and evolutions in biomedical technology, progress has been made in these systems. This new therapeutic strategy may offer potential benefits for the treatment of patients with GB, which remains an incurable and dismal disease with insufficient therapeutic response to conventional therapies. However, studies concerning the majority of NPs targeting GB did not progress beyond animal model testing, mainly due to the current lack of evidence regarding drug safety, potential mid- to long-term toxicity, immunogenicity, and pharmacokinetic and pharmacodynamic profiling.

Several types of NPs were developed and tested in vitro and in vivo using GB models. The diversity of NPs in terms of size, shape, surface charge, and surface components renders these nanomaterials highly versatile. Distinct NP properties influence their capacity for crossing the BBB, their serum half-life, and their ability to encapsulate certain types of drugs.

The most promising NPs reported to date are the hybrid polymer nanoformulations with ligands for receptors present in glioma cells due to their biochemical versatility, combining different properties of diverse nanomaterials in one nanoformulation. Inorganic NPs, like GNP and SPION, also have some advantages. These NPs act not only as drug delivery systems but can also be used as radiosensitizers to ameliorate the effectiveness of radiotherapy, characterize lesions with MRI, and induce hyperthermia, therefore acting as nanotheranostics. Another important application of NPs is the delivery of siRNA to downregulate the expression of target genes with protumor activity, as well as other immune-mediating particles that can evoke a shift in the inherently immunosuppressed tumor microenvironment towards a microenvironment more prone to targeting by the immune system.

As research in nanotechnology-based drug delivery systems progresses and shows promising results, the investment in NP manufacturing will naturally increase, allowing the advancement from preclinical to clinical trials and from phase I to phase III clinical trials.

Despite all the advances made in the most recent years regarding the treatment and management of GB patients, we believe that there is a categorical need to further develop newer therapeutic modalities, namely nanoparticles, as there is a clear limitation to what can be achieved with a maximal safe resection of GB and conventional adjuvant chemoradiotherapy, owing to the highly heterogeneous and unstable genetic profile of these tumors, making tumor recurrence and disease progression a certainty.

## Figures and Tables

**Table 1:** Nanoparticle-based delivery systems that have entered clinical trials for the treatment of GB.

Name	Drug/Mechanism of Action	Coating	NP Type	Administration Route	Relevant References
**NanoTherm^®^**	Thermal ablation via alternating magnetic field	Aminosilane	SPION	Intratumour injection	[61]
**NU-0129**	Apoptosis inducer via Bcl2L12 targeting	Spherical nucleic acid	GNP	Intravenous injection	[62]
**Onyvide^®^**	Irinotecan +/− TMZ	PEG	Liposome	Intravenous injection/ convection-enhanced delivery	[63]
**Caelyx^®^**	Doxorubicin	PEG	Liposome	Intravenous injection	[64]
**2B3-101**	Doxorubicin + Gluthatione	PEG	Liposome	Intravenous injection	[65]
**C225-ILs-dox**	Doxorubicin + Anti-EGFR activity	Cetuximab	Liposome	Intravenous injection	[66]
**SGT-53**	Wildtype p53 sequence	Anti-TfR	Liposome	Intravenous injection	[67]

Anti-TfR: anti-transferrin receptor. EGFR: epidermal growth factor receptor. GNP: gold nanoparticle. PEG: polyethylene glycol. SPION: superparamagnetic iron oxide nanoparticles.

## References

[1] Alphandéry E. (2020). Nano-Therapies for Glioblastoma Treatment. Cancers.

[2] Shergalis A., Bankhead A., Luesakul U., Muangsin N., Neamati N. (2018). Current challenges and opportunities in treating glioblastoma. Pharmacol. Rev..

[3] Zhao M., van Straten D., Broekman M.L., Préat V., Schiffelers R.M. (2020). Nanocarrier-based drug combination therapy for glioblastoma. Theranostics.

[4] Arifin D.Y., Lee K.Y.T., Wang C.-H., Smith K.A. (2009). Role of Convective Flow in Carmustine Delivery to a Brain Tumor. Pharm. Res..

[5] Seystahl K., Wick W., Weller M. (2016). Therapeutic options in recurrent glioblastoma—An update. Crit. Rev. Oncol..

[6] Tan A.C., Ashley D.M., López G.Y., Malinzak M., Friedman H.S., Khasraw M. (2020). Management of glioblastoma: State of the art and future directions. CA Cancer J. Clin..

[7] Stupp R., Mason W.P., van den Bent M.J., Weller M., Fisher B., Taphoorn M.J., Belanger K., Brandes A.A., Marosi C., Bogdahn U. (2005). Radiotherapy plus concomitant and adjuvant te-mozolomide for glioblas-toma. N. Engl. J. Med..

[8] Stupp R., Taillibert S., Kanner A., Read W., Steinberg D.M., Lhermitte B., Toms S., Idbaih A., Ahluwalia M.S., Fink K. (2017). Effect of tumor-treating fields plus maintenance temozolomide vs maintenance temozolomide alone on survival in patients with glioblastoma: A randomized clinical trial. JAMA.

[9] Rabha B., Bharadwaj K.K., Pati S., Choudhury B.K., Sarkar T., Kari Z.A., Edinur H.A., Baishya D., Atanase L.I. (2021). Development of Polymer-Based Nanoformulations for Glioblastoma Brain Cancer Therapy and Diagnosis: An Update. Polymers.

[10] Pardridge W.M. (2007). Blood–brain barrier delivery. Drug Discov. Today.

[11] Faraji A.H., Wipf P. (2009). Nanoparticles in cellular drug delivery. Bioorganic Med. Chem..

[12] Ruiz-Garcia H., Ramirez-Loera C., Malouff T.D., Seneviratne D.S., Palmer J.D., Trifiletti D.M. (2021). Novel Strategies for Nanoparticle-Based Radiosensitization in Glioblastoma. Int. J. Mol. Sci..

[13] Droop A., Bruns A., Tanner G., Rippaus N., Morton R., Harrison S., King H., Ashton K., Syed K., Jenkinson M.D. (2018). How to analyse the spatiotemporal tumour samples needed to investigate cancer evolution: A case study using paired primary and recurrent glioblastoma. Int. J. Cancer.

[14] Tamura R., Miyoshi H., Sampetrean O., Shinozaki M., Morimoto Y., Iwasawa C., Fukaya R., Mine Y., Masuda H., Maruyama T. (2019). Visualization of spatiotemporal dynamics of human glioma stem cell invasion. Mol. Brain.

[15] Comba A., Faisal S.M., Varela M.L., Hollon T., Al-Holou W.N., Umemura Y., Nunez F.J., Motsch S., Castro M.G., Lowenstein P.R. (2021). Uncovering Spatiotemporal Heterogeneity of High-Grade Gliomas: From Disease Biology to Therapeutic Implications. Front. Oncol..

[16] Gregory J.V., Kadiyala P., Doherty R., Cadena M., Habeel S., Ruoslahti E., Lowenstein P.R., Castro M.G., Lahann J. (2020). Systemic brain tumor delivery of synthetic protein nanoparticles for glioblastoma therapy. Nat. Commun..

[17] Alghamri M.S., Banerjee K., Mujeeb A.A., Mauser A., Taher A., Thalla R., McClellan B.L., Varela M.L., Stamatovic S.M., Martinez-Revollar G. (2022). Systemic Delivery of an Adjuvant CXCR4–CXCL12 Signaling Inhibitor Encapsulated in Synthetic Protein Nanoparticles for Glioma Immunotherapy. ACS Nano.

[18] Chang Y., Cai X., Syahirah R., Yao Y., Xu Y., Jin G., Bhute V.J., Torregrosa-Allen S., Elzey B.D., Won Y.-Y. (2023). CAR-neutrophil mediated delivery of tumor-microenvironment responsive nanodrugs for glioblastoma chemo-immunotherapy. Nat. Commun..

[19] Zhang P., Rashidi A., Zhao J., Silvers C., Wang H., Castro B., Ellingwood A., Han Y., Lopez-Rosas A., Zannikou M. (2023). STING agonist-loaded, CD47/PD-L1-targeting nanoparticles potentiate antitumor immunity and radiotherapy for glioblastoma. Nat. Commun..

[20] Kozielski K.L., Ruiz-Valls A., Tzeng S.Y., Guerrero-Cázares H., Rui Y., Li Y., Vaughan H.J., Gionet-Gonzales M., Vantucci C., Kim J. (2019). Cancer-selective nanoparticles for combinatorial siRNA delivery to primary human GBM in vitro and in vivo. Biomaterials.

[21] Afzal O., Altamimi A.S.A., Nadeem M.S., Alzarea S.I., Almalki W.H., Tariq A., Mubeen B., Murtaza B.N., Iftikhar S., Riaz N. (2022). Nanoparticles in Drug Delivery: From History to Therapeutic Applications. Nanomaterials.

[22] Park K. (2014). Controlled drug delivery systems: Past forward and future back. J. Control Release.

[23] Matsumura Y., Maeda H. (1986). A new concept for macromolecular therapeutics in cancer chemo-therapy: Mechanism of tumoritropic accumulation of proteins and the antitumor agent smancs. Cancer Res..

[24] Wu J. (2021). The Enhanced Permeability and Retention (EPR) Effect: The Significance of the Concept and Methods to Enhance Its Application. J. Pers. Med..

[25] Aldea M., Florian I.A., Kacso G., Craciun L., Boca S., Soritau O. (2016). Nanoparticles for Targeting Intratumoral Hypoxia: Exploiting a Potential Weakness of Glioblastoma. Pharm. Res..

[26] Ortega-Berlanga B., Gonzalez C., Navarro-Tovar G. (2021). Recent Advances in the Use of Lipid-Based Nanoparticles Against Glioblastoma Multiforme. Arch. Immunol. Ther. Exp..

[27] Rajora M.A., Ding L., Valic M., Jiang W., Overchuk M., Chen J., Zheng G. (2017). Tailored theranostic apolipopro-tein E3 porphyrin-lipid nanoparticles target glioblastoma. Chem. Sci..

[28] Liu Z., Ji X., He D., Zhang R., Liu Q., Xin T. (2022). Nanoscale Drug Delivery Systems in Glioblastoma. Nanoscale Res. Lett..

[29] Zhang Y., Zhang L., Hu Y., Jiang K., Li Z., Lin Y.Z., Wei G., Lu W. (2018). Cell-permeable NF-κB inhibitor-conjugated liposomes for treatment of glioma. J. Control Release.

[30] Melnick K., Dastmalchi F., Mitchell D., Rahman M., Sayour E.J. (2022). Contemporary RNA Therapeutics for Gli-oblastoma. Neuromolecular Med..

[31] Zhu Y., Liang J., Gao C., Wang A., Xia J., Hong C., Zhong Z., Zuo Z., Kim J., Ren H. (2021). Multifunctional ginsenoside Rg3-based liposomes for glioma targeting therapy. J. Control Release.

[32] Hu Y., Jiang K., Wang D., Yao S., Lu L., Wang H., Song J., Zhou J., Fan X., Wang Y. (2022). Core-shell lipoplexes inducing active macropinocytosis promote intranasal delivery of c-Myc siRNA for treatment of glioblastoma. Acta Biomater..

[33] Scioli Montoto S., Muraca G., Ruiz M.E. (2020). Solid Lipid Nanoparticles for Drug Delivery: Pharmacological and Biopharmaceutical Aspects. Front. Mol. Biosci..

[34] Zhang J., Zhu X., Jin Y., Shan W., Huang Y. (2014). Mechanism study of cellular uptake and tight junction opening mediated by goblet cell-specific trimethyl chitosan nanoparticles. Mol. Pharm..

[35] Yu Z., Fan W., Wang L., Qi J., Lu Y., Wu W. (2019). Effect of surface charges on oral absorp-tion of intact solid lipid nanoparticles. Mol. Pharm..

[36] Saraiva C., Praça C., Ferreira R., Santos T., Ferreira L., Bernardino L. (2016). Nanoparticle-mediated brain drug delivery: Overcoming blood-brain barrier to treat neurodegenerative diseases. J. Control Release.

[37] Ak G., Ünal A., Karakayalı T., Özel B., Selvi Günel N., Hamarat Şanlıer Ş. (2021). Brain-targeted, drug-loaded solid lipid nanoparticles against glioblastoma cells in culture. Colloids Surf. B Biointerfaces.

[38] Mukherjee A., Waters A.K., Kalyan P., Achrol A.S., Kesari S., Yenugonda V.M. (2019). Lipid-polymer hybrid nano-particles as a next-generation drug delivery platform: State of the art, emerging technologies, and perspectives. Int. J. Nanomed..

[39] Shi K., Zhou J., Zhang Q., Gao H., Liu Y., Zong T., He Q. (2015). Arginine-Glycine-Aspartic Acid-Modified Li-pid-Polymer Hybrid Nanoparticles for Docetaxel Delivery in Glioblastoma Multiforme. J. Biomed. Nanotechnol..

[40] Ahmed S., Alhareth K., Mignet N. (2020). Advancement in nanogel formulations provides controlled drug re-lease. Int. J. Pharm..

[41] Zhang H., Zhai Y., Wang J., Zhai G. (2016). New progress and prospects: The application of nanogel in drug de-livery. Mater. Sci. Eng. C Mater. Biol. Appl..

[42] Nguyen T.T.T., Zhang Y., Shang E., Shu C., Torrini C., Zhao J., Bianchetti E., Mela A., Humala N., Mahajan A. (2020). HDAC inhibitors elicit metabolic reprogramming by targeting super-enhancers in glioblastoma models. J. Clin. Investig..

[43] Song P., Song N., Li L., Wu M., Lu Z., Zhao X. (2021). Angiopep-2-Modified Carboxymethyl Chitosan-Based pH/Reduction Dual-Stimuli-Responsive Nanogels for Enhanced Targeting Glioblastoma. Biomacro-Molecules.

[44] Songbo M., Lang H., Xinyong C., Bin X., Ping Z., Liang S. (2019). Oxidative stress injury in doxorubicin-induced cardiotoxicity. Toxicol. Lett..

[45] Javed B., Zhao X., Cui D., Curtin J., Tian F. (2021). Enhanced Anticancer Response of Curcumin- and Piper-ine-Loaded Lignin-g-p (NIPAM-co-DMAEMA) Gold Nanogels against U-251 MG Glioblastoma Multi-forme. Biomedicines.

[46] Henna T.K., Raphey V.R., Sankar R., Shirin V.K.A., Gangadharappa H.V., Pramod K. (2020). Carbon nanostructures: The drug and the delivery system for brain disorders. Int. J. Pharm..

[47] Mehra N.K., Palakurthi S. (2016). Interactions between carbon nanotubes and bioactives: A drug delivery per-spective. Drug Discov. Today.

[48] Salazar A., Pérez-de la Cruz V., Muñoz-Sandoval E., Chavarria V., García Morales M.L., Espinosa-Bonilla A., Sotelo J., Jiménez-Anguiano A., Pineda B. (2021). Potential Use of Nitrogen-Doped Carbon Nanotube Spong-es as Payload Carriers Against Malignant Glioma. Nanomaterials.

[49] Villegas J.C., Álvarez-Montes L., Rodríguez-Fernández L., González J., Valiente R., Fanarraga M.L. (2014). Multi-walled carbon nanotubes hinder microglia function interfering with cell migration and phagocytosis. Adv. Healthc. Mater..

[50] Pinel S., Thomas N., Boura C., Barberi-Heyob M. (2019). Approaches to physical stimulation of metallic nano-particles for glioblastoma treatment. Adv. Drug Deliv. Rev..

[51] Khursheed R., Dua K., Vishwas S., Gulati M., Jha N.K., Aldhafeeri G.M., Alanazi F.G., Goh B.H., Gupta G., Pau-del K.R. (2022). Biomedical applications of metallic nanoparticles in cancer: Current status and future perspectives. Biomed. Pharmacother..

[52] Jafari S., Derakhshankhah H., Alaei L., Fattahi A., Varnamkhasti B.S., Saboury A.A. (2019). Mesoporous silica na-noparticles for therapeutic/diagnostic applications. Biomed. Pharmacother..

[53] Bielecki P.A., Lorkowski M.E., Becicka W.M., Atukorale P.U., Moon T.J., Zhang Y., Wiese M., Covarrubias G., Ravichandran S., Karathanasis E. (2021). Immunostimulatory silica nanoparticle boosts innate immunity in brain tumors. Nanoscale Horiz..

[54] Zhu J., Zhang Y., Chen X., Zhang Y., Zhang K., Zheng H., Wei Y., Zheng H., Zhu J., Wu F. (2021). Angiopep-2 modified lipid-coated mesoporous silica nanoparticles for glioma targeting therapy overcoming BBB. Biochem. Biophys. Res. Commun..

[55] Ferreira-Gonçalves T., Ferreira D., Ferreira H.A., Reis C.P. (2021). Nanogold-based materials in medicine: From their origins to their future. Nanomedicine.

[56] Norouzi M. (2020). Gold Nanoparticles in Glioma Theranostics. Pharmacol. Res..

[57] Allen N.C., Chauhan R., Bates P.J., O’Toole M.G. (2022). Optimization of Tumor Targeting Gold Nanoparticles for Glioblastoma Applications. Nanomaterials.

[58] Coluccia D., Figueiredo C.A., Wu M.Y., Riemenschneider A.N., Diaz R., Luck A., Smith C., Das S., Ackerley C., O’Reilly M. (2018). Enhancing glioblastoma treatment using cisplatin-gold-nanoparticle conjugates and targeted delivery with magnetic resonance-guided focused ultrasound. Nanomedicine.

[59] Marekova D., Turnovcova K., Sursal T.H., Gandhi C.D., Jendelova P., Jhanwar-Uniyal M. (2020). Potential for Treatment of Glioblastoma: New Aspects of Superparamagnetic Iron Oxide Nanoparticles. Anticancer Res..

[60] Zhu X.M., Yuan J., Leung K.C., Lee S.F., Sham K.W., Cheng C.H., Au D.W., Teng G.J., Ahuja A.T., Wang Y.X. (2012). Hollow superparamagnetic iron oxide nanoshells as a hydrophobic anticancer drug carrier: Intracelluar pH-dependent drug release and enhanced cytotoxicity. Nanoscale.

[61] Maier-Hauff K., Ulrich F., Nestler D., Niehoff H., Wust P., Thiesen B., Orawa H., Budach V., Jordan A. (2010). Efficacy and safety of intratumoral thermotherapy using magnetic iron-oxide nanoparticles combined with external beam radiotherapy on patients with recurrent glioblastoma multiforme. J. Neuro-Oncol..

[62] Kumthekar P., Ko C.H., Paunesku T., Dixit K., Sonabend A.M., Bloch O., Tate M., Schwartz M., Zuckerman L., Lezon R. (2021). A first-in-human phase 0 clinical study of RNA interference–based spherical nucleic acids in patients with recurrent glioblastoma. Sci. Transl. Med..

[63] Clarke J.L., Molinaro A.M., Cabrera J.R., DeSilva A.A., Rabbitt J.E., Prey J., Drummond D.C., Kim J., Noble C., Fitzgerald J.B. (2017). A phase 1 trial of intravenous liposomal irinotecan in patients with recurrent high-grade glioma. Cancer Chemother. Pharmacol..

[64] Beier C.P., Schmid C., Gorlia T., Kleinletzenberger C., Beier D., Grauer O., Steinbrecher A., Hirschmann B., Brawanski A., Dietmaier C. (2009). RNOP-09: Pegylated liposomal doxorubicine and prolonged temozolomide in addition to radiotherapy in newly diagnosed glioblastoma—A phase II study. BMC Cancer.

[65] Mehrabian A., Dadpour S., Mashreghi M., Zarqi J., Askarizadeh A., Badiee A., Arabi L., Moosavian S.A., Jaafari M.R. (2023). The comparison of biodistribution of glutathione PEGylated nanoliposomal doxorubicin formulations prepared by pre-insertion and post-insertion methods for brain delivery in normal mice. IET Nanobiotechnol..

[66] Kasenda B., König D., Manni M., Ritschard R., Duthaler U., Bartoszek E., Bärenwaldt A., Deuster S., Hutter G., Cordier D. (2022). Targeting immunoliposomes to EGFR-positive glioblastoma. ESMO Open.

[67] Kim S., Harford J.B., Moghe M., Slaughter T., Doherty C., Chang E.H. (2019). A tumor-targeting nanomedicine carrying the p53 gene crosses the blood–brain barrier and enhances anti-PD-1 immunotherapy in mouse models of glioblastoma. Int. J. Cancer.

[68] Aparicio-Blanco J., Sanz-Arriazu L., Lorenzoni R., Blanco-Prieto M.J. (2020). Glioblastoma chemotherapeutic agents used in the clinical setting and in clinical trials: Nanomedicine approaches to improve their efficacy. Int. J. Pharm..

